# Unilateral approach to bilateral middle cerebral artery aneurysms: a large series and a proposed grading system to predict technical difficulties

**DOI:** 10.1007/s10143-025-03634-7

**Published:** 2025-06-12

**Authors:** Servet Inci, Sacide Kalaycioglu

**Affiliations:** Department of Neurosurgery, Medical Faculty, Hacettepe University, Ankara, Turkey

**Keywords:** Bilateral aneurysms, Contralateral approach, Grading system, Middle cerebral artery, Mirror aneurysms, Unilateral approach

## Abstract

This study includes the surgical treatment of 31 patients with bilateral MCA (bMCA) aneurysms using a unilateral approach and is the second largest series in the literature. The main aim of this study is to present a proposed grading system to preoperatively predict the difficulties in this approach. The clinical files, radiological studies, operation records, intraoperative video recordings, complications and outcomes of 31 patients were retrospectively reviewed. In the first part of this study (2001–2010), we operated on 22 patients with bMCA aneurysms and were able to clip the contralateral aneurysm via unilateral approach in only 12 cases (54.5%). Considering our experience from the surgery of these patients, we identified 5 parameters that reveal surgical difficulties. In the second part of the study (2011–2023), considering these 5 parameters, 20 patients were selected for unilateral approach and bilateral aneurysms could be clipped in 19 patients (95%). As a result, 26 of 31 patients (83.9%) were discharged with a favorable outcome (mRS 0–2, excellent-good). Intraoperative rupture occurred in one patient. Based on our experience, we developed a grading system with a total of 10 points by assigning points according to the degree of difficulty of each parameter and classified surgical difficulty as follows: 0–1 low difficulty, 2–3 moderate difficulty, 4–6 high difficulty and 7–10 very high difficulty. Patients with bMCA aneurysms can be operated via unilateral approach in experienced hands in properly selected cases. This grading system may help the neurosurgeon in this selection by preoperatively predicting intraoperative difficulties. This study is a completely retrospective file scan. This is not a clinical trial. Clinical trial number: not applicable.

## Introduction

Bilateral middle cerebral artery (bMCA) aneurysms is a special subgroup among multiple intracranial aneurysms, accounting for approximately 1% of all intracranial aneurysms [20, 30]. This rate increases up to 5% in centers where aneurysm surgery is frequently performed [10, 17, 28].

The best surgical management of bMCA aneurysms is still controversial. Various surgical options are available, including unilateral approach in selected cases [2, 9, 10] or single stage bilateral approaches [5, 13, 32]. Two-stage approaches can also be performed, but there is the risk of bleeding from unruptured aneurysm in the early or late postoperative period [15, 21, 30, 31, 34]. We present the second largest series in the literature, including 31 cases with bMCA aneurysms operated on through a unilateral approach.

**Fig. 1:**
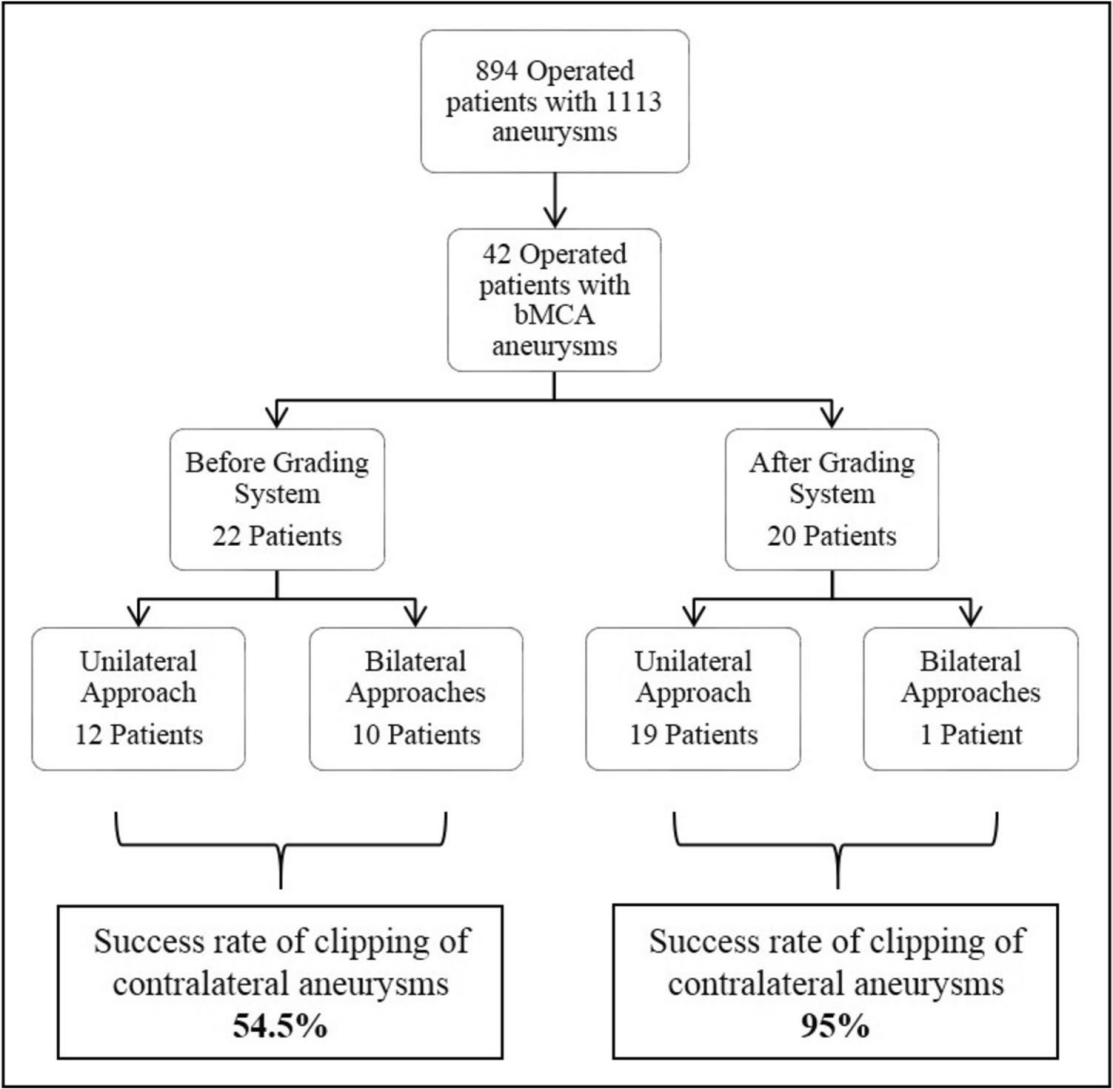
A flow diagram shows patient selection process

In 2012, we published our preliminary surgical experience regarding the approach to bMCA aneurysms in 22 cases [17]. We were able to clip the contralateral aneurysm via unilateral approach in only 12 of these 22 cases (54.5%). In the remaining 10 cases, it was necessary to perform bilateral craniotomy. We retrospectively analyzed by examining neuroradiologic studies and intraoperative findings of all 22 patients and determined 5 parameters for the feasibility of the unilateral approach to bMCA aneurysms: 1) The severity of brain edema, 2) Total length of the contralateral (A1 + M1) segments, 3) Configuration of the contralateral aneurysm, 4) Contralateral ICA bifurcation angle, and 5) Projection of the contralateral aneurysm. In the following years (2011–2023), taking these parameters into account, we operated on 20 more patients with bMCA aneurysms using unilateral approach. We were able to clip the contralateral aneurysm in 19 patients (95%). A flow diagram shows patient selection process (Fig. 1).

To our knowledge, there is no grading system that can preoperatively predict the difficulties of unilateral approach to bMCA aneurysms. Whereas, preoperative assessment of surgical difficulty is important in order to avoid complications and unsuccessful approaches. Therefore, our aim is to develop a grading system based on preoperative radiologic parameters that can predict surgical difficulty in unilateral approach to bMCA aneurysms, taking into account our experience.

## Material/method

Until April 2023, 1113 intracranial aneurysms in 894 patients were clipped by senior author (SI) at Hacettepe University Hospital. Among these cases, 31 patients with bMCA aneurysms were operated on with a unilateral approach. Medical records, radiological studies, surgical difficulties, intraoperative video recordings, complications and follow-up evaluations of these 31 patients were retrospectively reviewed. Consent was obtained from all patients regarding the use of the data both before hospitalization and surgery.

Preoperative cranial computed tomography (CT), CT-angiography and digital subtraction angiography (DSA) were performed in all patients and magnetic resonance imaging (MRI) in some patients. After 2002, 3-dimensional reconstructions of these angiographies were also obtained (3D-CTA and 3D-DSA).

Using all radiological examinations of the patients, we evaluated all following features: brain edema, length of the contralateral A1 and M1 segments, size/shape and projection of the contralateral aneurysm, contralateral ICA bifurcation angle, morphology of the Sylvian fissure, size/diameter of the contralateral Sylvian vessels and calcification of the contralateral aneurysm. All radiological data were then compared with intraoperative video recordings.

Patients with SAH were operated on as soon as possible, if conditions were suitable. Craniotomy was always performed on the side of the ruptured aneurysm. In cases without SAH, it was performed on the side of the larger/complex aneurysm.

In all operations, the blood flows of the parent artery and adjacent arteries were checked by microvascular Doppler ultrasonography before and after clipping. Intraoperative video angiography was also used for this purpose in recent years. In the early postoperative period, control angiography (DSA or CT-Angiography) was performed in all patients.

- **Olfactory function assessment**: Sense of smell of all the patients except three in moderate or poor condition was evaluated with several test substances (coffee, lemon, peppermint, etc.) in the early postoperative period and 1.5 to 6 months after surgeries. Patients were asked to identify these substances by smelling them while their eyes were closed. This procedure was performed by closing each nostril of the patient separately. Patients were asked to compare their current sense of smell with the preoperative one. If the patient said “I can smell less than before surgery”, this was considered as hyposmia, and if the patient said “I cannot smell at all”, this was considered as anosmia.

### Outcome

The outcome of the patients with was evaluated using the modified Rankin Scale (mRS) score at discharge from hospital and the last follow-up visit. These evaluations were carried out by neurosurgery residents/young neurosurgeons who did not attend any of the surgeries in this study. An mRS score of 0–2 was defined as a favorable outcome, whereas an mRS score of 3–6 was accepted as an unfavorable outcome.

### Surgical technique

**Fig. 2:**
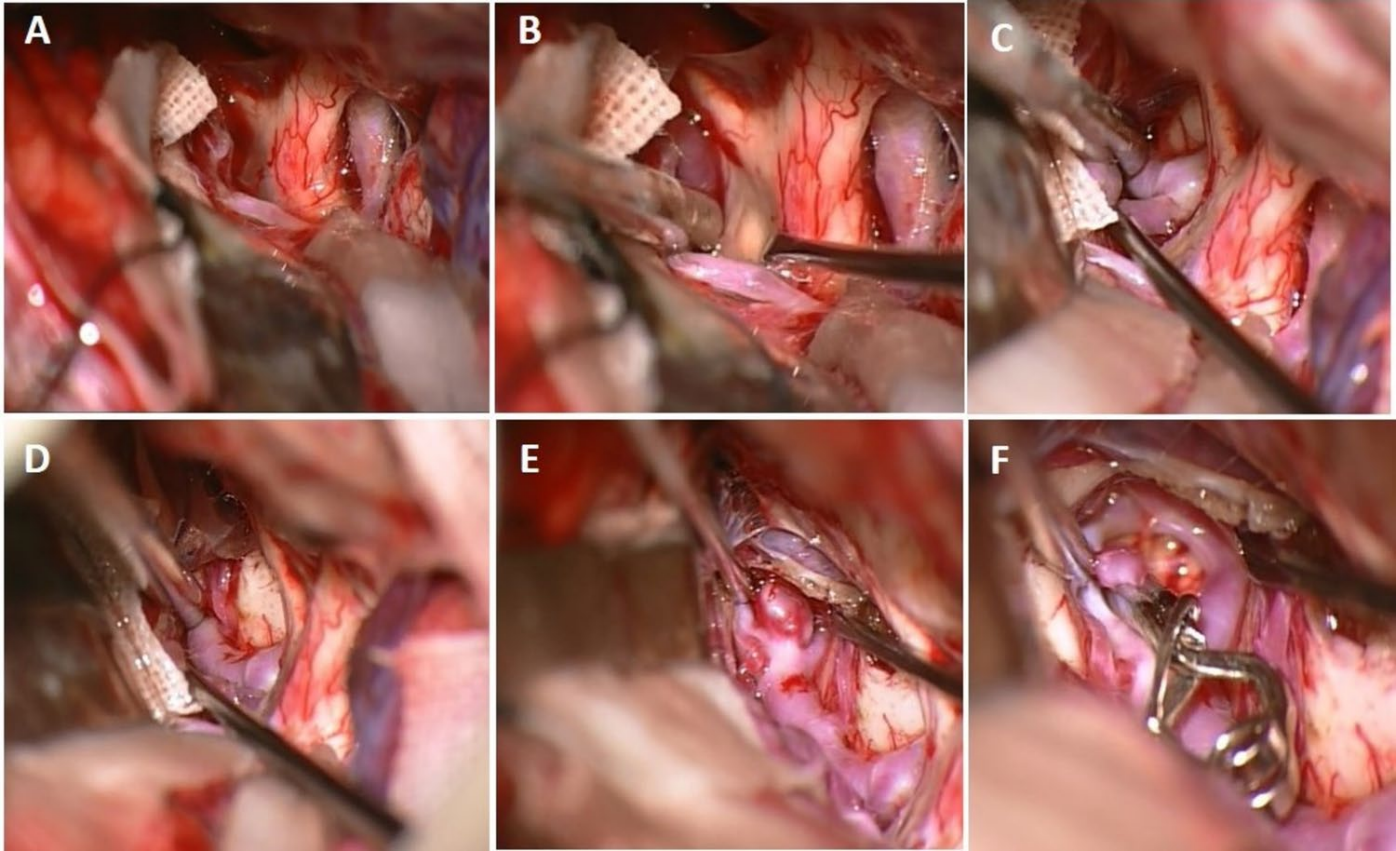
Basic surgical steps for unilateral approach to the contralateral MCA aneurysm. Details are in the “Surgical Technique” section

**Fig. 3:**
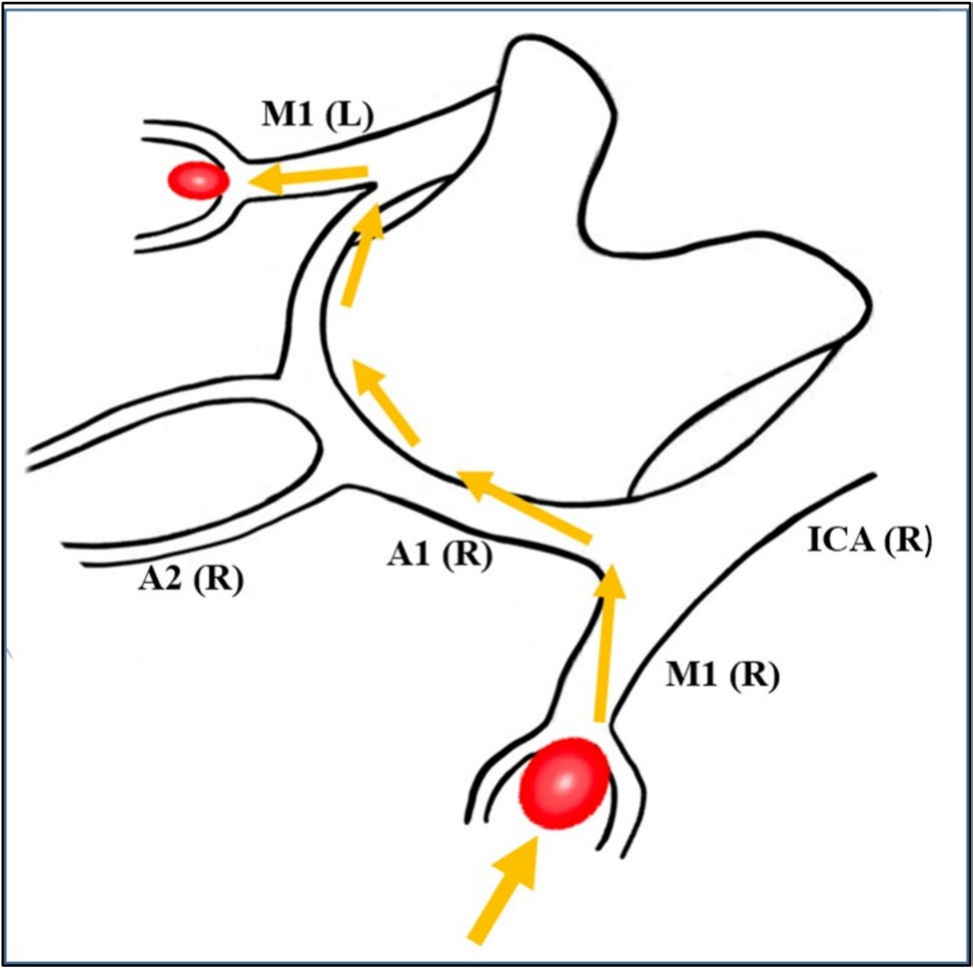
Artistic sketch shows basic surgical steps for unilateral approach to the contralateral MCA aneurysm

The head is rotated 20 to 25 degrees to the opposite side of the craniotomy and extended slightly to minimize frontal lobe retraction. Mannitol is routinely administrated at the beginning of the operation. Pterional craniotomy is always performed on the side of the ruptured aneurysm. The sphenoid wing and frontal base prominences are smoothed with high-speed drill. The Sylvian fissure is widely opened under the microscope and enough cerebrospinal fluid (CSF) is drained for slack brain. The ruptured aneurysm is clipped first using standard technique. The olfactory nerve is early identified and meticulously dissected from base of the frontal lobe. Arachnoidal trabeculations between frontal lobe and optic nerves are incised. Ipsilateral A1 segment is followed with microdissection towards ACoA complex (Fig. 2A). If necessary, the lamina terminalis is fenestrated (Fig. 2B) releasing CSF to slacken the brain for less frontal lobe retraction. The contralateral A1 segment is followed. When the contralateral ICA bifurcation is reached, the Sylvian cistern is opened and the proximal M1 segment is exposed (Fig. 2C). At this stage, the microscope is slightly rotated laterally to better visualization of the contralateral M1 segment (Fig. 2D). Larger Sylvian veins are preserved. Temporary clip is used to decrease tension of the contralateral aneurysm, if necessary. The contralateral aneurysm is dissected (Fig. 2E) and clipped with an appropriate clip (Fig. 2F). After clipping of the contralateral aneurysm, micro-Doppler examination is routinely performed in all patients. Then, if available, intraoperative video angiography is performed. After meticulous hemostasis, the wound is closed as the usual fashion. Early postoperative DSA/CTA is routinely performed for all patients. Figure 3 shows a schematic drawing of the steps of unilateral approach to bMCA aneurysms.

### Parameters

**Fig. 4:**
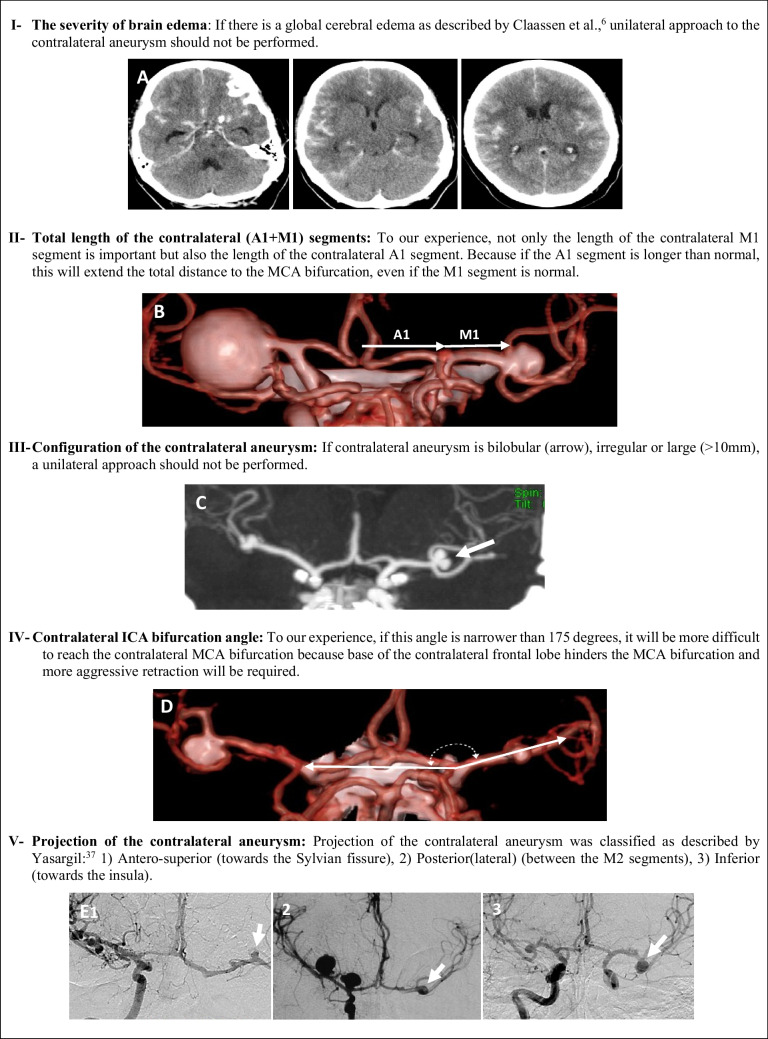
A brief summary of five parameters that predict the difficulties of unilateral approach to bMCA aneurysms

Technical difficulties encountered during surgery were retrospectively analyzed by examining all neuroimaging studies and intraoperative findings of all patients, and some surgical-radiological parameters were obtained for the feasibility of the unilateral approach to bMCA aneurysms. A brief summary of these five parameters is presented in Fig. 4.

- **Parameter I (The severity of brain edema)**: We have examined preoperative cranial CT of all patients and classified brain edema as no/focal or global using the method described by Claassen et al. [6]. If the following both findings were present, this condition was considered as global edema: 1) complete/near complete effacement of the hemispheric sulci and basal cisterns and 2) bilateral and extensive disruption hemispheric gray-white matter junction (Fig. 4A).

- **Parameter II (Total length of the contralateral A1 + M1 segments)**: To our experience, not only the length of the contralateral M1 segment is important but also the length of the contralateral A1 segment. Because if the A1 segment is longer than normal, this will extend the total distance from the midline to the MCA bifurcation, even if the length of M1 segment is normal. These lengths were measured by 3D-CT angiography and DSA after correction for magnification (Fig. 4B). Total length of (A1 + M1) was determined as short (< 35 mm), medium (35–40 mm) and long (> 40 mm).

- **Parameter III (Configuration of the contralateral aneurysm)**: Shape and size of the contralateral aneurysm were also evaluated by 3D-CT angiography and DSA after correction for magnification. The size of the contralateral aneurysm was evaluated as small (< 5 mm), medium (5–7 mm) and large (8–10 mm). Larger than 10 mm and non-saccular contralateral aneurysms were not operated on with a unilateral approach (Fig. 4C).

- **Parameter IV (Contralateral ICA bifurcation angle)**: To our experience, we defined an angle to assess another difficulty in unilateral approach and named it “Contralateral ICA Bifurcation Angle.” This angle was measured on the antero-posterior angiogram (Fig. 4D). The vertex (endpoint) of this angle is the bifurcation of the contralateral ICA. Ray (beam) A starts from the vertex and extends horizontally to ipsilateral side because the surgeon almost moves horizontally from the ipsilateral ICA bifurcation to contralateral ICA bifurcation. Ray B starts from the vertex and extends to the neck of contralateral MCA aneurysm because the surgeon has to follow this route after contralateral ICA bifurcation. We thought that if this angle gradually narrows, reaching to contralateral MCA bifurcation will be more difficult because base of the contralateral frontal lobe hinders the MCA bifurcation and more aggressive retraction will be required.

- **Parameter V (Projection of the contralateral aneurysm)**: This parameter was classified on the antero-posterior angiogram as described by Yasargil [37] (Fig. 4E): 1) antero-superior projection (towards the Sylvian fissure), 2) posterior (lateral) projection (between the M2 segments) and 3) inferior projection (towards the insula).

## Results

Clinical and demographic data of 31 patients with bMCA aneurysms and the characteristics of the aneurysms are shown in Table 1. In addition, all parameters of 31 patients who were operated via a unilateral approach are also presented in Table 2. All parameters of 10 patients who were operated via bilateral approach are also presented in Table 3.

**Table 1:** Characteristics of 31 patients and contralateral aneurysms

		%
Total number of the patients	31	
Age (years)	Mean 53.6 (range 43–66)	
Sex	Female 19 Male 12	61.3 38.7
Presentation	SAH 17 Incidental 14	54.8 45.2
Hunt&Hess Grade	I 4 II 7 III 6	23.5 41.2 35.3
Exact localization of the contralateral MCA aneurysm	MCA bifurcation 24 M1 7	77.4 22.6
Severity of cerebral edema*	No or Focal 31 Global 0	
Total length of the contralateral (A1 + M1) segments (mm)	Mean 30.2 (range 22–41) < 35 24 35–40 5 > 40 2	77.4 16.1 6.5
Size of the contralateral aneurysm (mm)	Mean 5.3 (range 3–10) < 5 mm 8 5–7 mm 21 8–10 2	25.8 67.7 6.5
Contralateral ICA bifurcation angle (degree)	Mean 178.8 (range 163–208) ≥ 175 19 < 175 12	61.3 38.7
Projection of the contralateral aneurysm**	Antero-superior 12 Posterior (lateral) 10 Inferior 9	38.7 32.3 29.0
Surgical complications	Intraoperative rupture 1 Anosmia/Hyposmia 3	3.2 9.6
Outcome***	Favorable 26 Unfavorable 3 Death 2	83.9 9.6 6.5
Follow-up (months)	Mean 42.4 (range 1–156)	

^*^According to Claassen classification,^6^ **According to Yasargil,^37^ ***mRS score

**Table 2:**
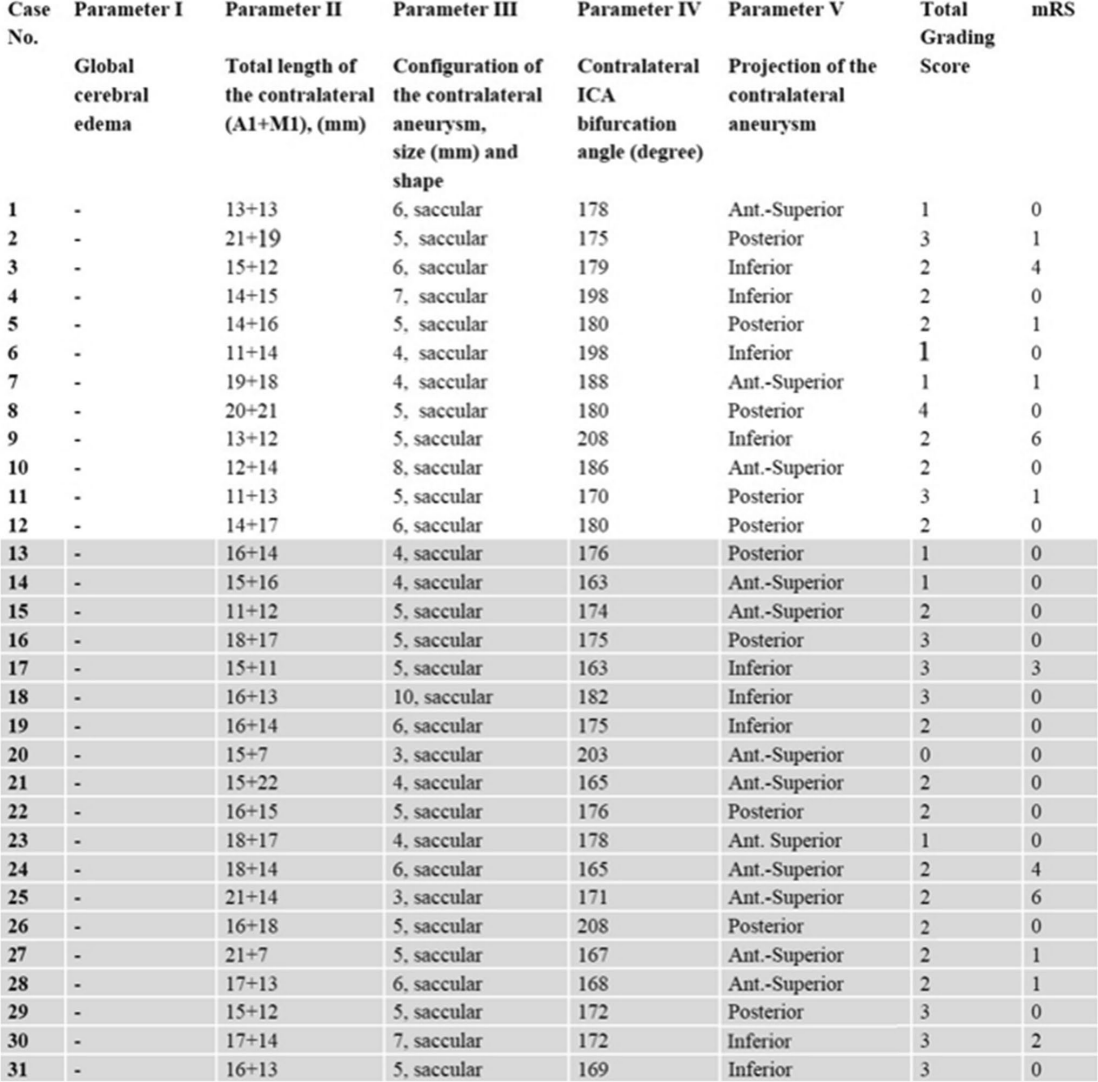
Parameters of 31 patients operated via unilateral approach

The parameters of the last 19 patients (gray background) were evaluated (after development of the grading system), and the parameters of the first 12 patients were evaluated retrospectively

**Table 3:** Parameters and outcomes of the patients operated on via bilateral craniotomy

Case No	Parameter I Global cerebral edema	Parameter II Total length of the contralateral (A1 + M1), (mm)	Parameter III Configuration of the contralateral aneurysm, size (mm) and shape	Parameter IV Contralateral ICA bifurcation angle (degree)	Parameter V Projection of the contralateral aneurysm	Total Grading Score	mRS
1	+	13 + 11	13, saccular	165	Posterior	8	3, moderate
2	-	20 + 27	7, saccular	205	Inferior	4	1, good
3	+	18 + 21	5, saccular	193	Inferior	7	0, excellent
4	+	20 + 26	14, saccular	166	Inferior	10	6, death
5	+	15 + 18	8, saccular	164	Ant.-Superior	7	0, excellent
6	-	16 + 19	13, saccular	194	Posterior	4	0, excellent
7	+	20 + 20	6, saccular	168	Posterior	8	3, moderate
8	+	15 + 13	7, saccular	180	Posterior	6	0, excellent
9	-	16 + 19	5, bilobular	246	Inferior	3	0, excellent
10	-	17 + 16	5, bilobular	185	Inferior	2	0, excellent

These parameters were evaluated retrospectively after the development of the grading system

Among these patients 19 were female (61.3%) and 12 were male (38.7%) with mean age of 53.6 years (range 43–66 years). Of the 31 patients, 17 (54.8%) presented with subarachnoid hemorrhage (SAH). In the remaining 14 patients (45.2%), the aneurysms were identified incidentally.

The preoperative condition of the patients with SAH was evaluated according to the Hunt&Hess grade. 4 patients presented with a grade I, 7 patients with grade II, and 6 patients with grade III. No grade IV patient underwent unilateral approach.

In all 17 patients with SAH, the ruptured aneurysms could be correctly identified by carefully examination of patients' neuroimaging studies. All contralateral aneurysms were unruptured, saccular and without wall calcification.

In this series, the average length of contralateral A1 segment was 15.8 mm (range 11–21 mm) and contralateral M1 segment was 14.4 mm (range 7- 22 mm). Contralateral aneurysm sizes measured between 3 and 10 mm (mean 5.3 mm). The mean contralateral ICA bifurcation angle was 178.8 degrees (range 163–208 degrees). The projections of the contralateral aneurysms were antero-superior in 12 patients, posterior (lateral) in 10 patients and inferior in 9 patients.

There were a total of 17 additional aneurysms in 31 patients: 5 ACoA aneurysms, 5 ipsilateral M1 aneurysms, 1 ipsilateral PCoA aneurysm, 1 ipsilateral ICA bifurcation aneurysm, 1 contralateral A2 aneurysm, 1 AChA aneurysm and 3 contralateral M1 aneurysms.

All contralateral aneurysms could be clipped with a single clip. Temporary clip was not used in 7 of 20 patients. The mean duration of temporary clip use in the other 13 patients was 4.7 min (range 2 to 18 min). Postoperative angiography showed complete occlusion of the aneurysms in all cases except one. In this case, a small remnant on contralateral aneurysm was seen on control angiogram and required an ipsilateral craniotomy.

### Complications

The most important complication was intraoperative rupture which developed in one patient (3.2%). Details of this case are presented in Illustrative Case 25.

In addition, anosmia developed in 1 patient (3.2%) and hyposmia developed in 2 patients (6.4%).

Clinical vasospasm developed in 6 patients. 4 of them received medical treatment. In the other 2 patients, balloon angioplasty and intra-arterial vasodilator agents (nimodipine, verapamil) were applied. Complications that developed in other patients did not affect the neurological status and outcomes of the patients (hydrocephalus in 3 patients, epidural hematoma in 1 patient, subdural effusion in 2 patients and wound infection in 1 patient). Complications data are presented as a dedicated table (Table 4).

**Table 4:** Detailed postoperative complications in 31 patients operated via unilateral approach

Complications	Patients (n)	%	Treatment
Anosmia	1	3.2	-
Hyposmia	2	6.4	-
Hydrocephalus	3	9.6	V/P shunt (1 patient)
Epidural Hematoma	1	3.2	Surgery
Subdural Effusion	2	6.6	No treatment was required
Wound Infection	1	3.2	Medical treatment and dressing
Clinical Vasospasm	6	19.3	Medical treatment and/or angioplasy

### Statistical analyses

**Fig. 5:**
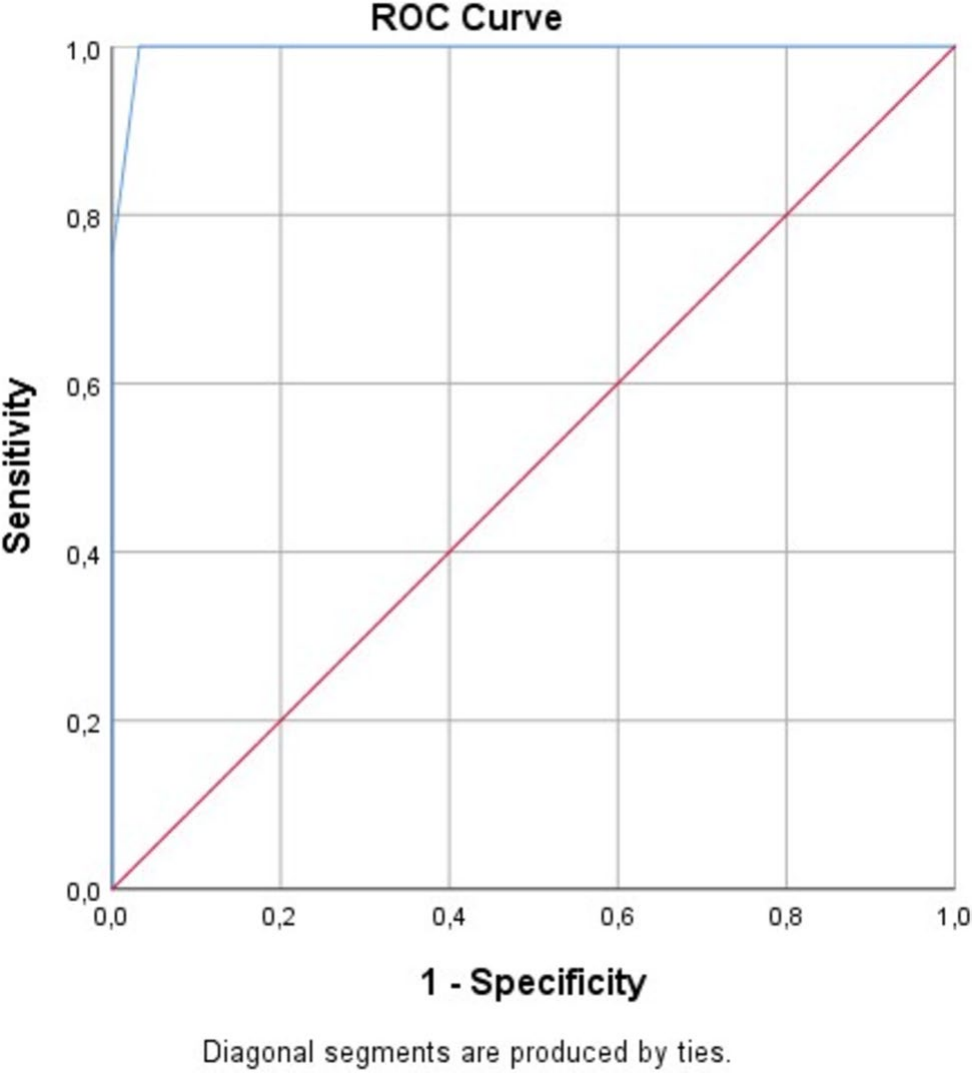
ROC analysis graph

- **Operation time**: In the unilateral group, the mean operation time (from skin to skin) was 250.9 min and 463.1 min in the bilateral group. This difference is statistically highly significant (P = 0,0001 Student’s t test). The mean operation time is approximately 46% shorter in the unilateral group than bilateral group. Moreover, operating time becomes shorter as more experience is gained.

- **Hospital stay**: In the unilateral group, the mean length of hospital stay (from admission to discharge) was 11.4 days and 18.0 days in the bilateral group. These results show that the unilateral approach provides a 37% shorter hospital stay. This difference is also statistically significant** (P = 0.005, Student’s t test).**

- **Total cost**: The mean total cost (from admission to discharge) per patient was US $7,617 for the unilateral group and US $9,932 for the bilateral group. That is the mean cost per patient is 23% lower in the unilateral group (**P = 0.026, Student’s t test**).

- **Mortality**: There was no mortality in the bilateral craniotomy group (10 patients). In the unilateral group (31 patients), there was 1 mortality. However, the difference was not statistically significant** (Chi-square test, p = 0.451).**

- **ROC analysis**: According to the Receiver Operating Characteristic (ROC) analysis results, the prediction of individuals switching to the bilateral method with the total score they received from the grading system (0–10) shows a near-perfect accuracy (AUC = 0.996, p < 0.001) (Fig. 5). The most appropriate cut-off point was determined as 4. At this threshold, the sensitivity of the test was calculated as 100% and the specificity as 96.8%. These findings indicate that the bilateral method should be strongly recommended in individuals with a total score of 4 and above.

- **Multi Variant Logistic Regression Analysis**: The agreement between parameter 1 (4 points) and the applied method was evaluated with **Cohen's Kappa test**. The results showed that there was a very high level of agreement in the method selection in these individuals (**κ = 0.827, p < 0.001**). This high agreement shows that parameter 1 can play a decisive role in clinical decisions and can strongly predict the method to be selected.

In the logistic regression analysis performed with the **forward stepwise method** after excluding parameter 1, parameter 3 (p = 0.002) and then parameter 2 (p = 0.008) made a statistically significant contribution in predicting the method selection. The contribution size of these two parameters was OR = 28.23 (parameter 3) and OR = 8.60 (parameter 2), respectively, indicating that the power to predict the method to be applied was quite high.

It was observed that parameters 4 and 5 did not make a statistically significant contribution to the model (p > 0.05).

### Outcome

Although the majority of patients presented with SAH (54.8%), 26 patients (83.9%) were discharged with a favorable outcome (mRS, 0–2) and 3 patients (9.6%) with an unfavorable outcome (mRS, 3–5). The unfavorable outcomes were directly related to poor preoperative grade (Hunt&Hess, Grade III) or subsequent vasospasm. There were two deaths in the postoperative period. As mentioned above, one patient died due to ischemic complications after intraoperative rupture (surgical mortality 3.2%) and another patient who was hospitalized with Hunt & Hess grade III and had preoperative vasospasm died due to severe vasospasm (3.2%). Clinical summary of 31 patients with bMCA aneurysms operated via unilateral approach and their outcomes of each patient are presented in the Table 5.

**Table 5:** Clinical summary of 31 patients with b-MCA aneurysms operated via unilateral approach

Case No	Age, Sex	Presentation	Ruptured (ipsilateral) aneurysm, size (mm)	Associated aneurysms, size (mm)	Clipping	Complication	mRS
1	44, F	SAH	MCA, R, 5	MCA, L, 6	Both	-	0, excellent
2	46, F	SAH	MCA, R, 8	MCA, L, 5	Both	Hydrocephalus, V/P shunt	1, good
3	64, M	SAH	ACoA	MCA, R, 6 MCA, L, 6	All	Vasospasm	4, poor
4	65, F	SAH	MCA, L, 13	MCA, R, 7	Both	-	0,excellent
5	46, M	SAH	MCA, L, 13	MCA, R, 5	Both	-	1, good
6	53, M	SAH	MCA, R, 15	MCA, L, 4	Both	-	0, excellent
7	45, F	SAH	MCA, R, 7	MCA, R, 4 M1, L, 4	All	-	1, good
8	47, F	SAH	MCA, L, 8	MCA, R, 5 M1, R, 4	All	-	0, excellent
9	65, F	SAH	MCA, R, 18	MCA, L, 5	All	Severe vasospasm	6, death
10	48, F	SAH	MCA, R, 15	MCA, L, 8 ACoA, 5	All	-	0, excellent
11	54, M	SAH	MCA, R, 12	MCA, L, 5	Both	-	1, good
12	45, F	SAH	MCA, L, 18	MCA, L, 6	Both	-	0, excellent
13	65, M	Incidental	MCA, L, 8	MCA, R, 4	Both	-	0, excellent
14	54, F	Incidental	MCA, R, 7	M1, R, 3 M1, L, 4	All	-	0, excellent
15	45, F	Incidental	MCA, R, 5	M1, R, 3 MCA, L, 4 ACoA	All	-	0, excellent
16	50, F	Incidental	MCA, R, 6	MCA, L, 5 M1, R, 3	All	-	0, excellent
17	64, M	SAH	MCA, R, 6	MCA, L, 5	Both	Residue in contralateral aneurysm, second craniotomy required	3, moderate
18	43, M	Incidental	MCA, R, 7	MCA, L, 5 A2, L, 3	Both	-	0, excellent
19	51, F	Incidental	MCA, R, 9	M1, L, 3 PCoA, R, 4	Both	-	0, excellent
20	48, F	Incidental	MCA, L, 8	M1, R, 3	Both	-	0, excellent
21	66, F	Incidental	MCA, L, 6	MCA, R, 3 M1, R, 3	Both	-	0, excellent
22	50, F	Incidental	MCA, R, 10	MCA, L, 4	Both	-	0, excellent
23	45, F	Incidental	MCA, 11, R	M1, L, 4 ACoA, 6 M1, R, 3	All		0, excellent
24	64, M	SAH	MCA, R, 9	MCA, L, 4	Both	Vasospasm	4, poor
25	61, F	Incidental	MCA, R, 5	M1, R, 3 ACoA, 7 M1, L, 3	All	Intraoperative rupture	6, death
26	64, F	Incidental	MCA, R, 10	MCA, L, 4	All	-	0, excellent
27	62, M	SAH	MCA, L, 8	MCA, R, 4 ACoA, 4	All	Epidural hematom	1, good
28	50, F	SAH	MCA, R, 7	MCA, L, 5	Both	-	1, good
29	49, M	SAH	MCA, R, 9	M1, L, 4 AChA, R, 4 ICA, R, 5	All	-	0, excellent
30	60, M	Incidental	MCA, R, 7	MCA, L, 3	Both	Anosmi	2, good
31	50, M	Incidental	MCA, R, 7	MCA, L, 4	Both	-	0, excellent

### Follow-up

Two patients were lost to follow-up. The other 27 surviving patients (93.1%) were followed for an average of 42.4 months (range 1–156 months). There was no rebleeding in any patient during the follow-up period. No patient worsened neurologically in this period of time.

### Proposed grading system

In light of our experience from all the patients (31 unilateral, 11 bilateral), we have developed a grading system for preoperative assessment of the technical difficulties of a unilateral approach to bMCA aneurysms. The proposed grading system was created by giving points to each of the 5 determined parameters according to their difficulty level: 1) the severity of brain edema (0 or 4 points), 2) total length of the contralateral (A1 + M1) segments (0, 1 or 2 points), 3) configuration of the contralateral aneurysm (0, 1 or 2 point), 4) contralateral ICA bifurcation angle (0 or 1 point) and finally 5) projection of the contralateral aneurysm (0 or 1 point). By adding the total points, a 10-points grading system was obtained (Grade 0–10) (Table 6).

**Table 6:** The proposed grading system to predict the difficulties encountered during unilateral approach to bMCA aneurysms

Parameters	Criteria	Points
I- Severity of brain edema	**No or Focal** **Global**	**0** **4**
II- Total length of the contralateral (A1 + M1) segments	** < 35 mm** **35-40 mm** ** > 40 mm**	**0** **1** **2**
III- Configuration (shape/size) of the contralateral aneurysm	**Saccular, Small** (< 5 mm) **Saccular, Medium** (5–7 mm) **Saccular, Large** (8–10 mm)	**0** **1** **2**
IV- Contralateral ICA bifurcation angle	** ≥ *175***^***o***^ ** < *175***^***o***^	**0** **1**
V- Projection of the contralateral aneurysm	**Antero-superior** **Other projections**	**0** **1**
TOTAL		**10**

According to our evaluation, the range of 0–1 points indicates low difficulty, 2–3 points indicates intermediate difficulty, 4–6 points indicates high difficulty, 7–10 points indicates very high difficulty (A unilateral approach should not be attempted)

Surgical difficulty is assessed by the neurosurgeon considering the following divisions: 0–1 low difficulty, 2–3 intermediate difficulty, 4–6 high difficulty (One must be very careful when deciding on a unilateral approach) and 7–10 very high difficulty (A unilateral approach should not be attempted).

In the second part of this study (2011–2023), considering these 5 parameters, 20 more patients with bMCA aneurysm were selected and operated on using unilateral approach. Thus, the predictive value of this grading system were tested. The contralateral aneurysms could be clipped in 19 of 20 patients (95%). Although the parameters were appropriate, we could not reach the contralateral aneurysm in only one patient with SAH due to dense arachnoid adhesions surrounding the contralateral M1 segment, and ipsilateral craniotomy was required.

### Illustrative cases

**Fig. 6:**
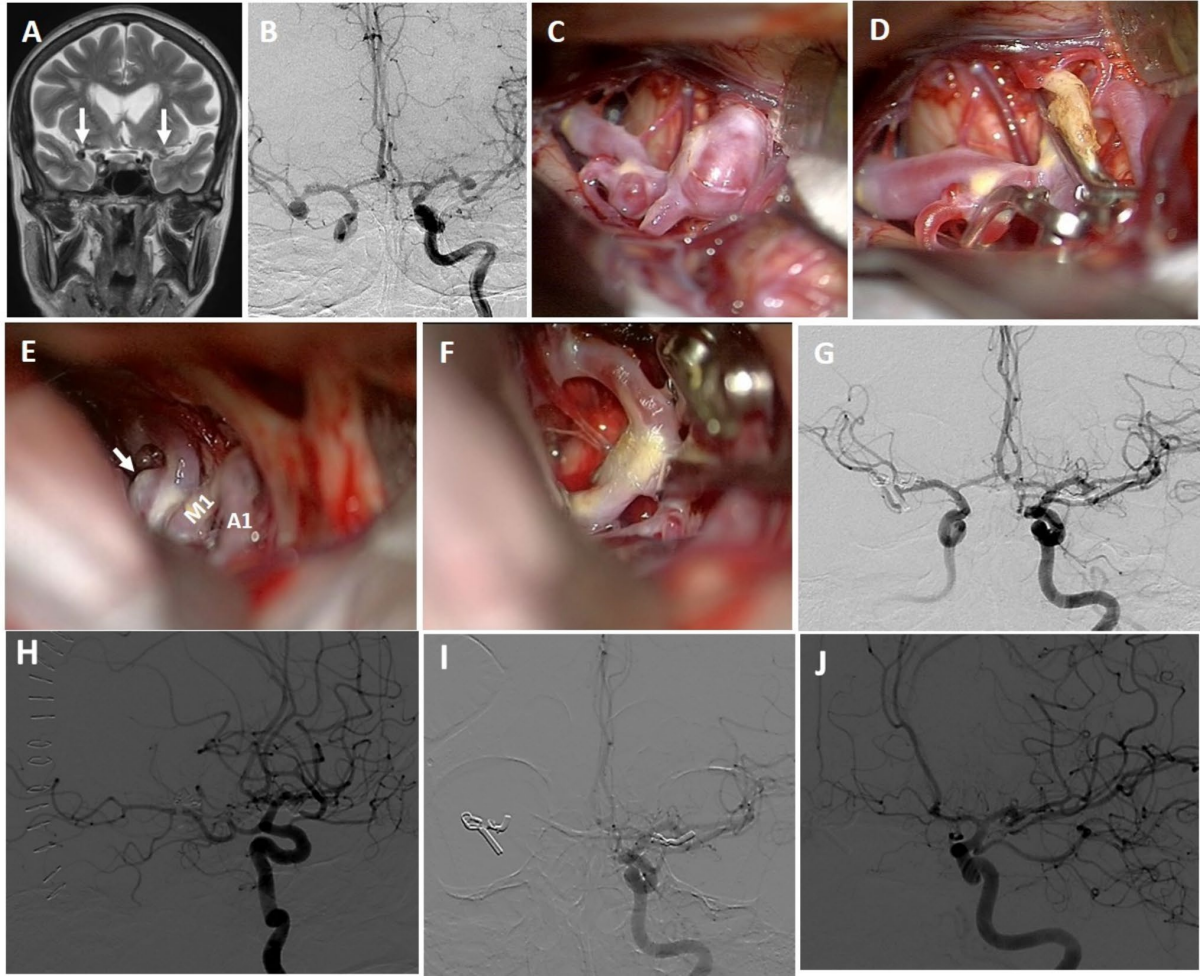
**A** T2-weighted MR image shows bilateral aneurysms (arrows), **B** DSA demonstrates three aneurysms: in the right MCA bifurcation and M1 segment and in the left MCA bifurcation, **C** At first, both aneurysms in the right MCA were explored, **D** were clipped, **E** and then right A1 and left A1 segments were followed and the left ICA bifurcation was reached. Tortuous left M1 segment was followed towards the bifurcation and a posterior (lateral) projection aneurysm was seen. **F** The perforating arteries at the bifurcation were dissected and the aneurysm was closed with a slightly bent clip passing under the inferior trunk. **G**, **H**, **I**, **J** Postoperative DSA confirms occlusion of three aneurysms

**Fig. 7:**
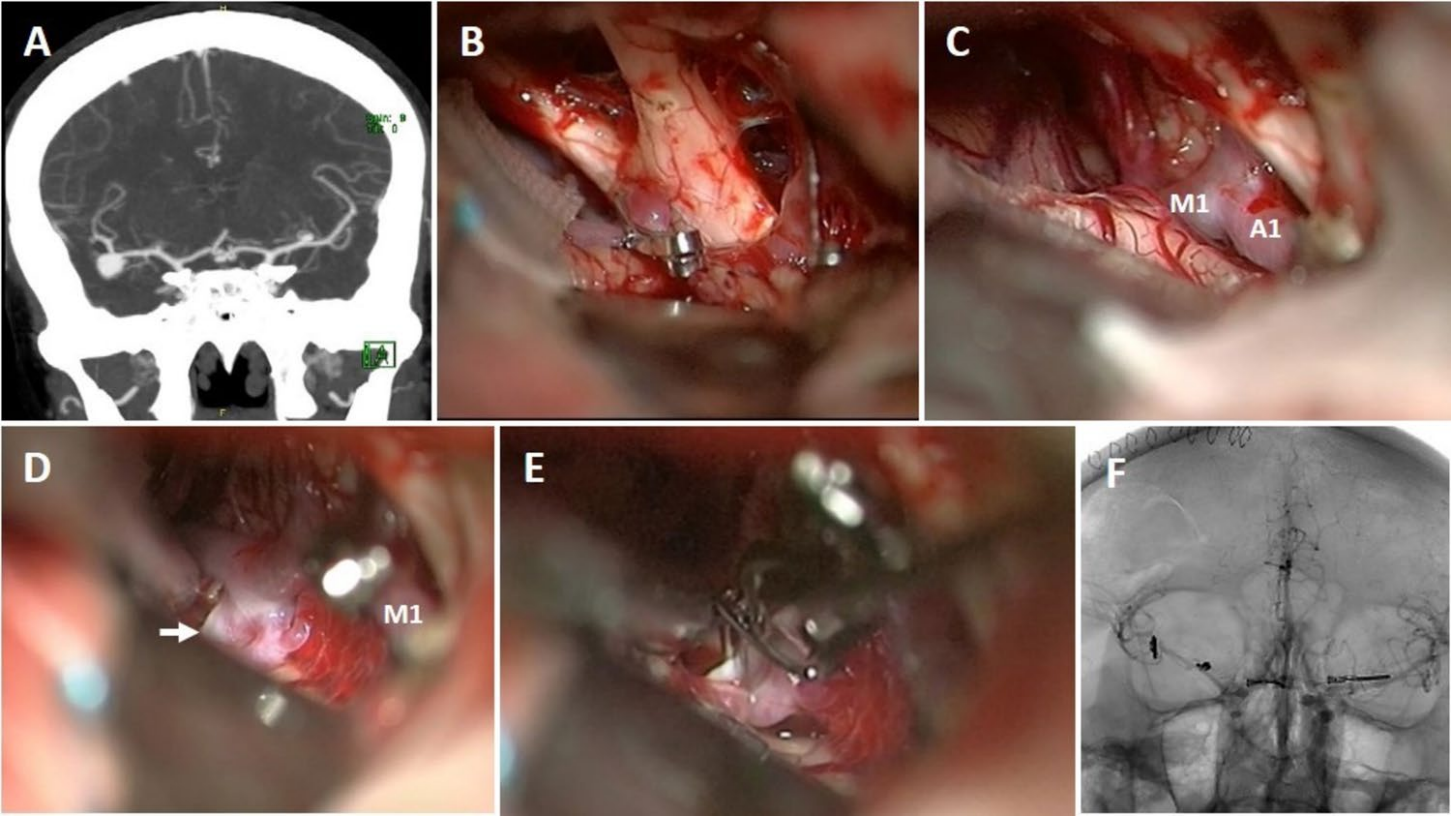
**A** Coronal section of CT-angiogram shows four aneurysms: right MCA bifurcation and M1 segment, ACoA and left M1 segment, **B** After two aneurysms in the right MCA were clipped, the ACoA aneurysm was explored and clipped with a small slightly curved clip, **C** The left ICA bifurcation was reached by following the left A1 segment, **D** the small aneurysm at the left M1-Anterior temporal artery junction was explored (arrow) using the temporary clip, **E** and clipped with a sideward clip, **F** control angiogram confirmed total occlusion of all four aneurysms

**Fig. 8:**
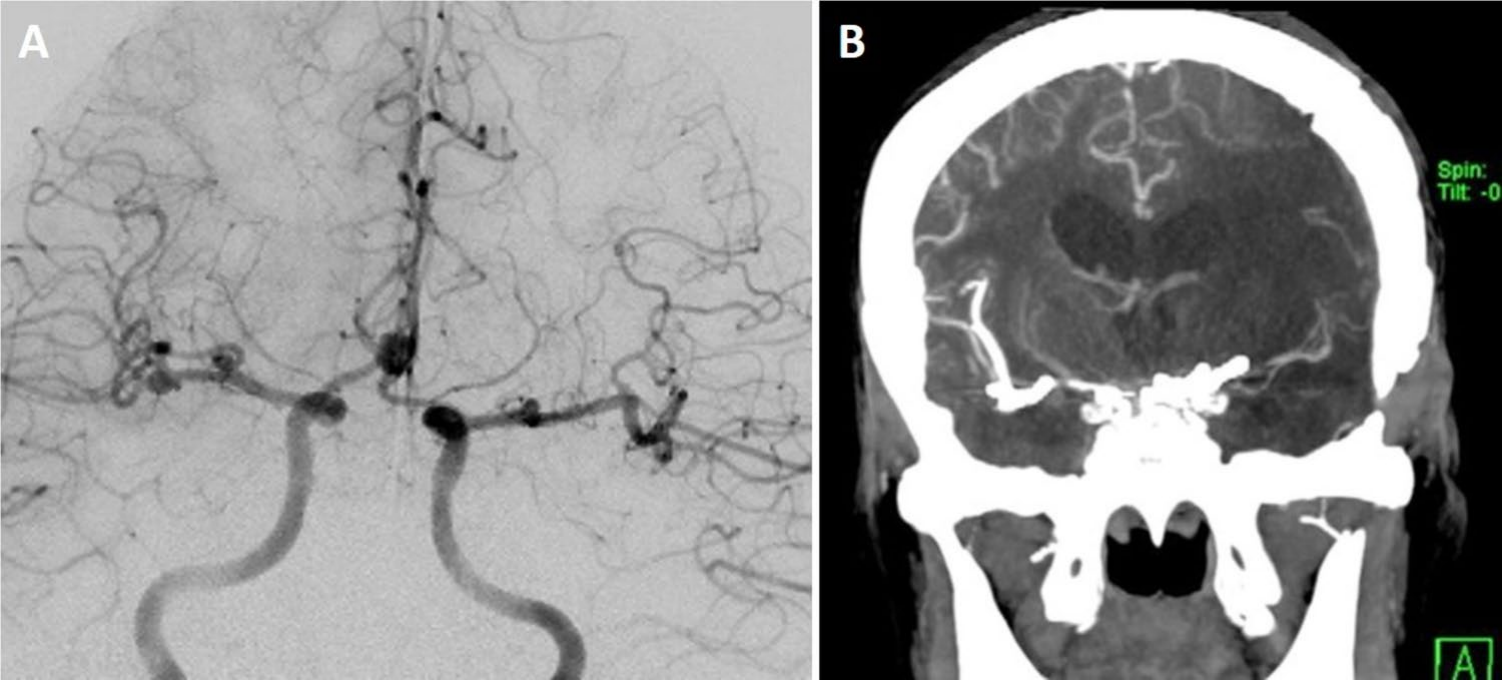
**A** DSA shows four aneurysms, **B** CT angiogram shows infarction in the MCA territory despite normal blood flow in the left MCA

- **Case 16**: A 50-year-old female patient was examined in another hospital due to atypical headache and was referred to our clinic after bilateral aneurysms were seen on MRI (Fig. 6A). Her neurological examination was normal. DSA showed total three aneurysms: in right MCA bifurcation, right M1 segment and left MCA bifurcation (Fig. 6B). According to the proposed grading system, the difficulty level of the surgery was evaluated as intermediate (size 1 point, total length 1 point, posterior projection 1 point). The patient was operated via right pterional approach. Both aneurysms in the right MCA were explored (Fig. 6C) and clipped (Fig. 6D) using standard technique. And then, the left ICA bifurcation was reached by following the right and left A1 segments. The partially tortuous left M1 segment was followed towards the MCA bifurcation by microdissection. The aneurysm with posterior (lateral) projection was seen (Fig. 6E). Its neck was prepared and clipped with a small bent clip passing under the inferior trunk of the M2 segment. (Fig. 6F). Postoperative angiogram showed complete occlusion of all aneurysms (Fig. 6G, H, I, J). The patient was discharged with normal neurological examination. At last follow-up (13 months), her neurologic examination was normal.

- **Case 23**: A 45-year-old female patient was investigated due to previous/suspected subarachnoid hemorrhage. CT angiogram revealed four aneurysms: large right MCA bifurcation aneurysm, small right M1 segment aneurysm, medium ACoA aneurysm and small left M1 segment aneurysm (Fig. 7A). According to the proposed grading system, the difficulty level of the surgery was evaluated as low (total length 1 point). The right pterional craniotomy was performed and large MCA bifurcation (previously ruptured) and small M1 aneurysms were explored and clipped. Later, ACoA aneurysm was explored and clipped (Fig. 7B). Subsequently, the left A1 segment was followed and the left ICA bifurcation was reached (Fig. 7C). The left M1 segment was followed and the aneurysm (arrow) was prepared using a temporary clip (Fig. 7D) and clipped with a small sideward clip (Fig. 7E). Postoperative angiogram demonstrated occlusion of all four aneurysms (Fig. 7F). The patient was discharged as neurologically intact. At last follow-up 119 months later, her neurological examination was still normal.

- **Case 25**: A 61-year-old hypertensive female patient was examined at another hospital due to chronic headache. Three aneurysms were detected in radiological examinations. The patient was referred to our clinic for further examination and treatment. Her neurological examination was normal. DSA showed four aneurysms at right MCA bifurcation (5 mm), right M1 (3 mm), ACoA (7 mm) and left M1 (3 mm) (Fig. 8A). Her grading score was 2 (total length 1 point, ICA bifurcation angle 1 point). Surgery was recommended to the patient and possible risks and complications were explained.

Right pterional craniotomy was performed. MCA bifurcation aneurysm, M1 aneurysm and subsequently ACoA aneurysm were clipped without any problem. The left A1 segment was followed and the contralateral ICA bifurcation was reached. By following the M1 segment, MCA bifurcation and the aneurysm were observed. An unexpected intraoperative rupture developed at the beginning of aneurysm dissection. Contralateral M1 segment was immediately occluded with a small temporary clip. While the blood was aspirated a large suction, the aneurysm neck was prepared with difficulty due to the lack of distal control and eventually clipped. Doppler examination showed that blood flow in the MCA was normal. But, unfortunately, the temporary clip usage time was 18 min. The patient was taken to the intensive care unit under anesthesia (without awakening) with anti-edema treatment (steroid and mannitol). In the early postoperative period, anisocoria developed. Cranial CT showed large infarct area in the MCA territory and slight midline shift. There was no time to do an angiography. The patient was immediately taken to the operating room and decompressive craniotomy and duraplasty were performed. The anisocoria recovered, but the patient's neurological condition did not improve. When the patient's condition stabilized, the bone flap was replaced and a control CT angiography was performed. Although blood flow was present in the MCA, infarct area was still visible (Fig. 8B). Most probably, infarction was due to long-term use of temporary clip (18 min.). Over time, tracheostomy and percutaneous endoscopic gastrostomy were required. Unfortunately, she died six months later because of pulmonary infection and eventually sepsis.

**Fig. 9:**
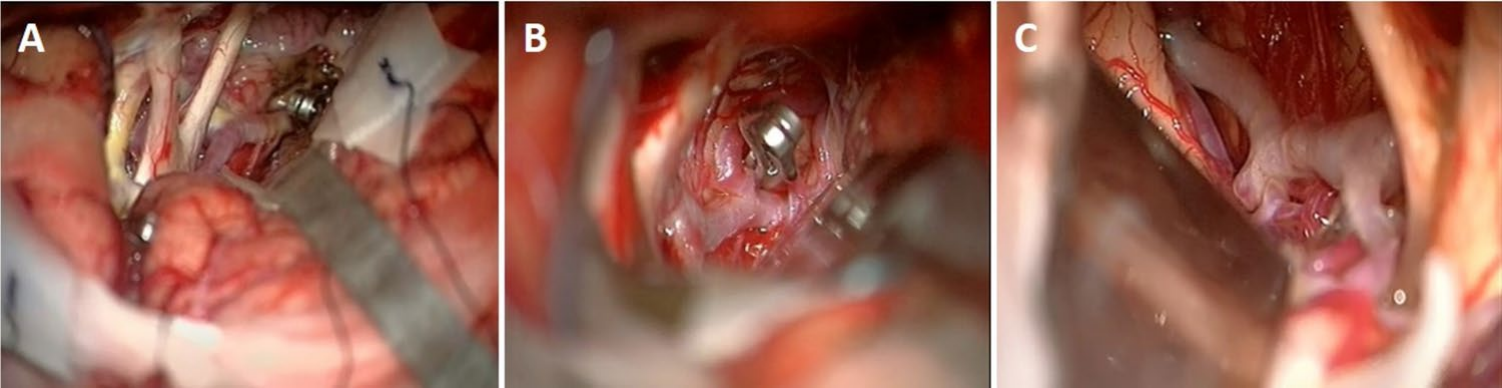
Intraoperative photographs of other 3 patients with bMCA aneurysms

Additionally, intraoperative photographs of the other 3 patients in this series are also shown in Fig. 9 A-B-C.

## Discussion

bMCA aneurysms represent a special subgroup within the bilateral-multiple supratentorial aneurysms because they have the longest surgical pathway. The patients with bMCA aneurysms can be treated with various surgical approaches (unilateral approach, bilateral approach in the same session, bilateral approaches in two stages) or endovascular methods. Several surgical series regarding unilateral approach to bMCA aneurysms have been published. Details of the main surgical series (≥ 5 cases) on this subject are shown in Table 7 [1, 2, 5, 9, 10, 16, 20, 25, 27, 29, 30, 35, 36, 38, 39]. If the medical condition of the patient and surgical parameters are appropriate, we also prefer the unilateral approach. There are some advantages and disadvantages of this approach.

**Table 7:** Surgical series containing 5 or more cases regarding unilateral approach to bMCA aneurysms in the literature

Author/Year	Number of patients with bMCA aneurysms approached unilaterally	Presentation (n)	Exact location of contralateral MCA aneurysm	Mean size of contralateral MCA aneurysms	Surgical Complications (%)
de Oliveira et al., 1996^9^	23	NS	All at M1	NS	None
McMahon et al. 2001^20^	6	SAH 2 Incidental 4	NS	NS	None
de Sousa et al., 2005^10^	30	SAH 28 Incidental 2	All at M1	All < 10 mm	None
Park et al., 2009^25^	6	SAH	All at bifurcation	All < 10 mm	Anosmia/hyposmia 66%
Santana Pereira et al., 2006^30^	11	NS	All at bifurcation	All < 10 mm	None
Rajesh et al., 2010^27^	5	All SAH	All at bifurcation	4 mm	None
Hopf et al., 2010^16^	10	SAH 5 Incidental 5	NS	NS	Anosmia/Hyposmia 20%
Rodriguez-Hernandez et al., 2012^29^	11	SAH 3 Incidental 8	All at bifurcation	3.7 mm	None
Andrade-Barazarte et al., 2015^2^	38	SAH 9 Incidental 29	Bifurcation 22 M1 16	3.8 mm	Anosmia/Hyposmia 21.0%
Yu J et al., 2016^38^	10	All SAH	NS	3.2 mm	NS
Wang et al., 2017^36^	8	SAH 3 Incidental 5	NS	NS	NS
Yu L–H et al., 2017^39^	5	SAH 3 Incidental 2	All at M1	7.3 mm	NS
Acik et al., 2017^1^	7	All SAH	Bifurcation 4 M1 3	7 mm	IOR 28.5% Anosmia/Hyposmia 14.2%
Cho et al., 2017^5^	19	All incidental	Bifurcation 17 M1 2	Mostly < 5 mm	IOR 5% Anosmia 58%
Wang et al., 2024^35^	16	NS	All at bifurcation	NS	Hyposmia 12,5%
Present series, 2025	31	SAH 17 Incidental 14	Bifurcation 24 M1 7	5.3 mm	IOR 3.1% Anosmia/Hyposmia 9.6%

*IOR* Intraoperative rupture, *NS* not specified

### Advantages and disadvantages

The main advantages of a unilateral approach to bMCA aneurysms are: 1) Operation time is approximately 42–46% shorter than bilateral approaches, [1, 2, 17] 2) Hospital stay is approximately 30–37% shorter, [1, 17] 3) Surgical cost is up to 23% lower, [17] 4) Cosmetic defect is less due to single craniotomy, 5) It allows aggressive vasospasm treatment when necessary, and 6) It eliminates of the risk of bleeding from unruptured aneurysm in the early or late postoperative period. In patients with multiple aneurysms, probability of bleeding from an unruptured aneurysm after clipping of the ruptured aneurysm is low, but many cases have been reported [15, 21, 30, 31, 34].

On the other hand, unilateral approach also has some disadvantages: 1) The surgical corridor is quite long, 2) The surgical corridor is cone-shaped and gradually narrows, so maneuverability is limited on the contralateral side, 3) There is a possibility that the olfactory nerve may be damaged, and 4) If intraoperative rupture occurs during dissection/clipping of contralateral aneurysm, its management is quite difficult.

### Preoperative nuances

**Fig. 10:**
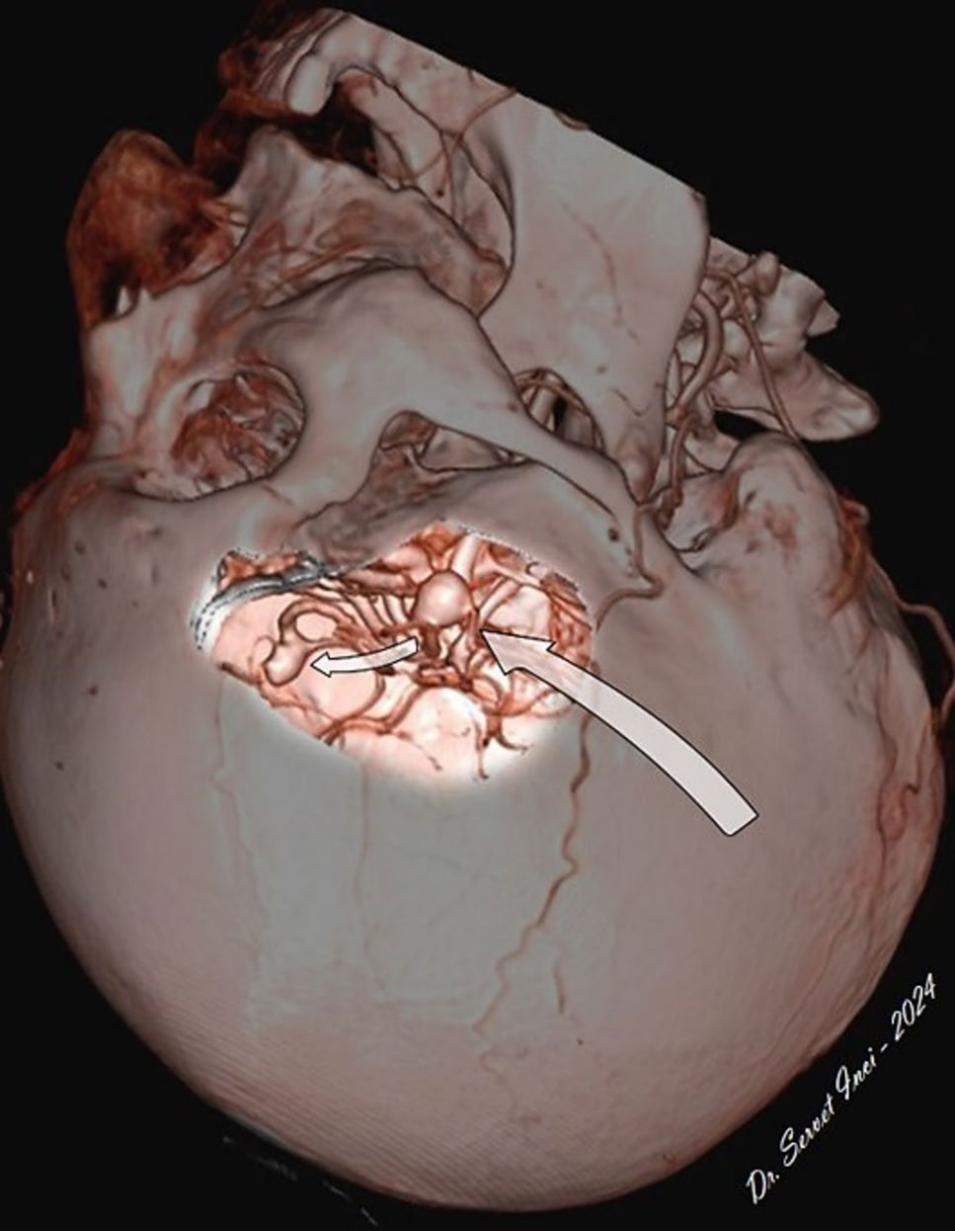
3D image of the skull, the intracranial vascular structures and the aneurysms after virtual unilateral pterional craniotomy. The neurosurgeon should use this fascinating technique

First of all, the neurosurgeon must visualize three-dimensional knowledge of the vascular structures in the surgical corridor in his/her mind, preoperatively. For this purpose, simulation images (3D-CTA or 3D-DSA) that can be rotated 360 degrees are extremely useful for accurate localization and projection of the contralateral aneurysm within the cranium from the surgeon's intraoperative perspective. In other words, 3D images should be used by the neurosurgeon as a"vascular map"within the cranium (Fig. 10).

In unilateral approach, the most important point in preoperative period is correct diagnosis of the side where the aneurysm ruptured, because unilateral craniotomy should always be performed on the ruptured side. Nowadays, the probability of misdiagnosis is low due to the advanced neuroimaging techniques. In a case series recently reported by Orning et al. [23], the overall misdiagnosis rate in identifying the source of hemorrhage in the presence of multiple aneurysms was 4.3%. Probability of misdiagnosis should be lower in bMCA aneurysms because the aneurysms are quite far from each other. Despite everything, if it is not possible to accurately identify the ruptured aneurysm, a craniotomy should be performed on the side where the aneurysm is larger and irregular.

Of course, every patient with bMCA is not suitable for unilateral approach. In addition to the parameters we have determined, the following factors should also be evaluated in terms of the feasibility of the unilateral approach using all neuroimaging studies: 1) the relationship of the contralateral aneurysm to the sphenoid wing, 2) diameter and number of the contralateral Sylvian veins. Large and numerous veins may prevent proper intraoperative visualization of the contralateral M1 segment and the aneurysm, 3) morphology of the contralateral Sylvian fissure (may be large, narrow or adherent), 4) calcification at the contralateral aneurysm neck that can make clipping difficult.

### Surgical tips and tricks

**Fig. 11:**
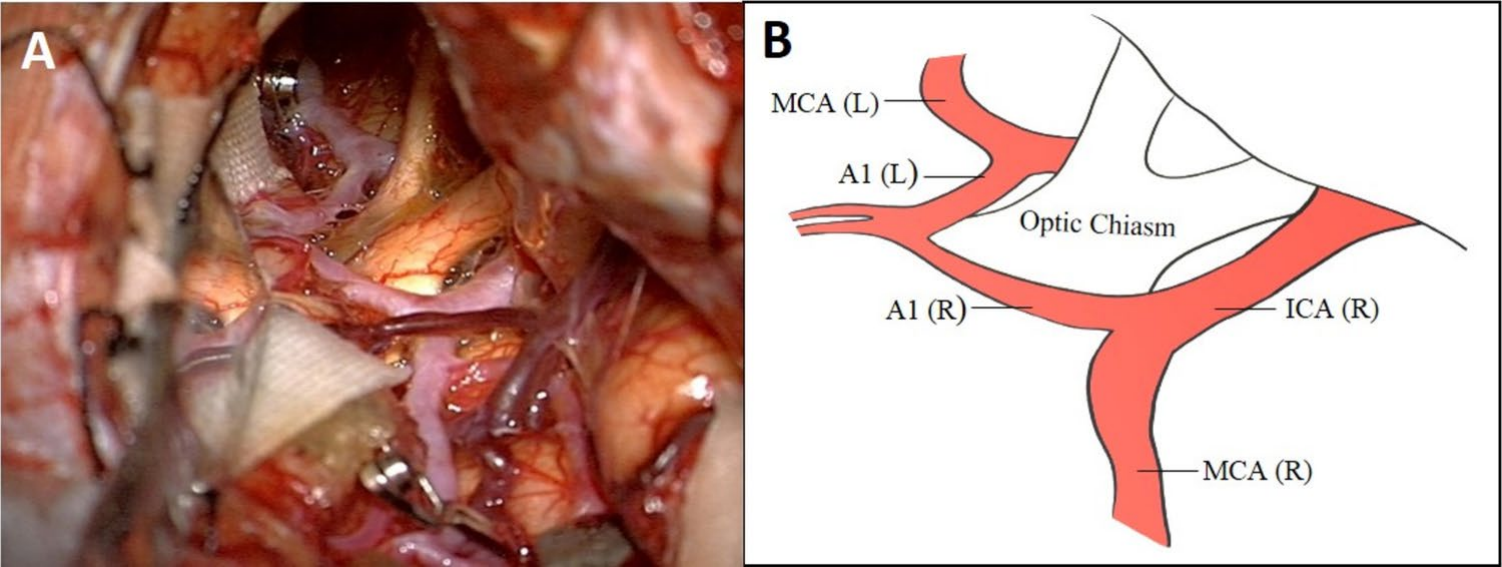
**A** Intraoperative photograph after clipping of bMCA aneurysms through the right pterional approach. The surgical corridor gradually narrows towards the contralateral aneurysm, **B** Similar artistic sketch of the same photograph

According to our experience, it would be useful for the neurosurgeons to pay attention to the following points in these technically difficult cases: 1) First of all, the neurosurgeon should know that, surgical corridor will gradually narrow and manipulations will become difficult even in patients without SAH (Fig. 11A-B). 2) Longer micro instruments are required to expose and clip the contralateral aneurysm and should therefore be readily available in the operating room. In most of these cases, standard microsurgical instruments (clip applicator, micro-dissector, micro-scissors) with a length of 19–21 cm are sufficient. In only 4 cases, that distance of contralateral (A1 + M1) segments was longer than 35 mm and in these cases, 1–2 cm longer instruments had to be used*,* 3) After ipsilateral Sylvian fissure is widely opened and the ruptured aneurysm is clipped, the olfactory nerve should be meticulously dissected from the frontal base to prevent olfactory dysfunction, 4) While contralateral Sylvian fissure opens from proximal to distal, large Sylvian veins should be preserved as much as possible because the results of venous sacrifice are not predictable, 5) Dissection should be performed as much as possible along the inferior surface of the M1 segment. This reduces the possibility of injury to the lateral lenticulostriate arteries, 6) As dissection proceeds distally along the contralateral M1 segment, the anterior temporal artery is a useful landmark for both surgical orientation and determining the distance to the MCA bifurcation, 7) If there is incomplete understanding of the relevant vascular anatomy during dissection, the neurosurgeon should review the patient's angiography and compare it with the existing surgical anatomy, 8) Temporary clip can be used during the dissection of the contralateral aneurysm to decrease tension in the dome and to facilitate neck dissection, but short or mini temporary clips should be used to avoid obstructing the surgeon's field of vision, 9) Rarely, there may be more than one aneurysm on contralateral MCA as in our two cases. In these cases, clip sequencing is very important. Which aneurysm should be clipped first? If proximal aneurysm is clipped first, the operative exposure is narrowed because of the clip head. Therefore, the necks of both aneurysms should be prepared properly before the first clip is placed and distal aneurysm should be clipped first, 10) If the neurosurgeon intraoperatively realizes that current conditions are not suitable for clipping the contralateral aneurysm, he/she should not continue fighting and opt for a second craniotomy, 11) If a craniotomy was mistakenly performed on the side where the aneurysm did not rupture, the operation should be terminated after clipping this aneurysm. No attempt should be made to clip the ruptured contralateral aneurysm from the same craniotomy. The ruptured aneurysm should be operated in the same session via second craniotomy, 12) It should be known that if there is a remnant in the contralateral aneurysm on control angiography (as in one of our cases mentioned in results section), this clip may not be easily removed during ipsilateral craniotomy. Because the head (lock) of the clip will remain in the opposite direction from the surgeon's view. Therefore, great care must be taken to avoid the remnant.

### Complications

The most important and feared complication of unilateral approach *is intraoperative rupture of the contralateral aneurysm*. Management of this complication is extremely difficult because of deep and narrow surgical area. Although it is easy to achieve proximal control by placing a temporary clip on the contralateral M1 segment, it is almost impossible to provide distal control. We experienced this terrible complication in one case. In this stressful condition, our recommendations for the neurosurgeons are as follows: 1) remain calm, because the movements of a neurosurgeon in panic lead to more serious bleeding and injury to neural tissues, 2) immediately use temporary clip for proximal control, 3) aspirate rapidly blood in the surgical field by a large suction, 4) advance aspiration toward the bleeding point by following the jet blood stream until the tear appears, 5) if the neck is not ready but a bleeding point is visible, place a small pilot clip over the aneurysm dome (just proximal to the rupture site) to stop the bleeding, 6) if this is not possible, put a small piece of cotton on the rupture point and gently press with the suction tube (the bleeding point itself is fragile and, therefore excessive pressure should not be applied), 7) quickly dissect the neck and clip the aneurysm, and finally ***8) do not forget: avoiding intraoperative rupture is always better than its management.***

Another known and annoying complication of the unilateral approach to bilateral aneurysms is *anosmia/hyposmia*. Its incidence is generally around 10 to 20% [1, 2, 16, 17]. In a series, this rate is quite high, [25] but the authors noted that no olfactory dissection was used during frontal lobe retraction. On this occasion, we compared our olfactory nerve injury rate in this series (9.6%, 3/31) with our rate in the last 31 single MCA aneurysm surgeries we performed (3.2%,1/31). Although this complication was numerically more in bilateral MCA group, this difference was not statistically significant (Chi-square test, p = 0.291). The most important reason for this is that the olfactory nerve is stretched and separated from the cribriform plate due to excessive retraction of the frontal lobe. To avoid this complication, early identification and dissection of the nerve is important. Arachnoidal dissection should be performed parallel to the nerve using sharp instruments to separate the olfactory nerve from the base of the frontal lobe. Mobilization of the nerve in this way allows retraction of the frontal lobe by 10 to 15 mm, resulting in a lower rate of olfactory dysfunction [3, 4, 7]. However, exploration and therefore dissection of the contralateral olfactory nerve is quite difficult. If the falx cerebri is cut over the crista galli, this procedure can be done, although difficult [4]. According to an experimental study, if the nerve continuity is preserved, dexamethasone treatment may have a therapeutic value [19]. Therefore, great care should be taken to preserve the nerve's continuity. Another cause of olfactory dysfunction is excessive and prolonged retractor pressure on the nerve itself. This can cause loss of function due to impaired vascular supply of the nerve. Loosening the retractor for 15–30 s every five minutes may reduce the risk of dysfunction [3]. If intermittent dynamic retraction with a suction tube (retractorless surgery) can be performed instead of a static brain retractor, this can reduce the risk of nerve damage. However, this may not be possible in every patient, especially when approaching the contralateral aneurysm.

### Parameters

**Fig. 12:**
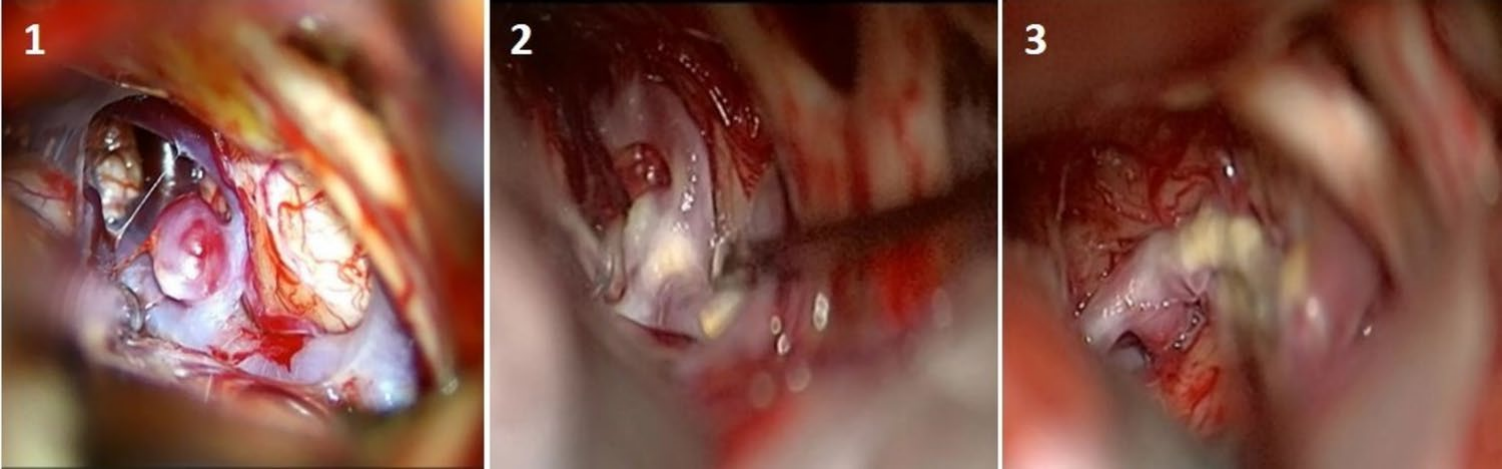
Intraoperative pictures of each projection of the contralateral aneurysms: 1) Antero-superior, 2) Posterior (lateral) 3) Inferior

I) Of course, the most important of 5 parameters we determined is *the severity of brain edema*. Previous publications also noted the importance of cerebral edema, [9, 10, 38] but did not use a precise classification. Such a clear classification defined by **Claassen et al.** [6] was used by us for the first time in our previous publication [17]**.** The presence of global brain edema makes very difficult microdissection, narrowing the surgical corridor and causing excessive brain retraction. Although CSF drainage from the Sylvian fissure, basal cisterns and lamina terminalis somewhat reduces intracranial pressure, approaching contralateral aneurysm is still very difficult because the brain itself is swollen. Therefore, if there is severe cerebral edema (red-angry brain) due to acute SAH on preoperative cranial CT, unilateral approach to contralateral MCA aneurysm is not recommended, at least one should be very careful in making the decision. In the first part of this study, we tried to reach the contralateral aneurysm in a patient with global cerebral edema, but we had to terminate it. We never attempted after that. Therefore, the highest score (4 points) was given to global cerebral edema in the proposed grading system. Multi Variant Logistic Regression Analysis results also confirmed that cerebral edema was the most important parameter.
II) In previous publications, the importance of the length of contralateral M1 segment has been emphasized [9, 10, 20, 24, 39]. To our experience, the length of the contralateral A1 is also important as M1 segment. Because if the A1 segment is longer than normal, this will extend the total distance, even if the length of M1 segment is normal. In this situation, the total distance from the midline to contralateral MCA bifurcation will also be longer than expected. The importance of *the total length of (A1* + *M1) segment* was emphasized by us for the first time in our previous report [17]. To our experience, if total length is shorter than 35 mm, the exploration of the contralateral MCA bifurcation is relatively easy (assigned difficulty score 0). If total length is between 35–40 mm, the exploration becomes more difficult, but is still possible (assigned difficulty score 1). If it is longer than 40 mm, the exploration is quite difficult (assigned difficulty score 2).
III) Configuration (shape and size) of the contralateral aneurysm is also important. Contralateral large and giant aneurysms should not be operated using unilateral approach because these aneurysms require some special techniques such as aneurysmorrhaphy, thrombectomy, multi-clipping techniques. It is extremely difficult and dangerous to apply these techniques in this narrow and deep surgical area. Therefore, only saccular aneurysms smaller than 10 mm should be considered in this approach. Contralateral aneurysms were divided into 3 groups according to their size: < 5 mm, 5–7 mm, 8–10 mm, and their difficulty degree evaluated as 0, 1 and 2 points, respectively. In addition, the shape of the aneurysm is also important besides its size. If the contralateral MCA aneurysm is bilobular, it should not be operated unilaterally because there is a high probability of remnant remaining.
IV) Contralateral ICA bifurcation angle: To our experience, if this angle is narrower than 175 degrees, it will be more difficult to reach the contralateral MCA bifurcation because base of the contralateral frontal lobe hinders the MCA bifurcation and more aggressive retraction will be required. Therefore, 0 points were given for an angle wider than 175 degrees and 1 point for an angle narrower than 175 degrees.
V) Projection of the contralateral aneurysm: 1) In *antero-superior projection* (towards the Sylvian fissure), the dome may be adherent to the arachnoid of the Sylvian cistern. Rarely, the medial orbital gyrus may hide these aneurysms and resection of a small portion of the gyrus may be required. Clipping is usually relatively easy with a straight clip, 2) In *posterior (lateral) projection*, the dome projects along the axis of MCA and the neck often incorporated superior or inferior trunk or both. Therefore, clipping is little more difficult [1, 2, 9, 10, 39]. Usually a curved clip is required for occlusion of the neck, 3) In *inferior projected aneurysms*, the M1 segment regularly covers the neck region, so the M1 trunk itself may hinder visualization of the neck. In addition, lenticulostriate arteries may be in close relation to the aneurysm base and their visualization is of great importance before bipolar reshaping of the dome and clipping of the neck. Therefore, exploration of the neck is more difficult [16, 39]. As a result, to our experience, clipping of antero-superior aneurysms is easier than the other projections [9, 10].Therefore, 0 points were given for supero-anterior projection and 1 point for other projections. Intraoperative pictures of each projection of the contralateral aneurysm are presented in Fig. 12.

In conclusion, the first 3 parameters (brain edema, total length and configuration) are the most important factors in deciding the feasibility of the unilateral approach (Multi Variant Logistic Regression Analysis). The fourth and fifth parameters (angle and projection) are not determining factors, but useful for calculating the difficulty of the unilateral approach.

### Rationale of this grading system

In previous publications, some factors that may affect the clipping of the contralateral MCA aneurysm have been highlighted, such as brain edema, length of contralateral M1 segment, contralateral aneurysm configuration [2, 10, 29]. However, none of these have provided a grading system that can objectively guide the neurosurgeon. In other words, there is no grading system in the literature predicting the surgical difficulties of a unilateral approach to bMCA aneurysms. We hypothesized that a simple and accurate grading system might predict surgical difficulties before surgery, guide the neurosurgeon in clinical decision-making and be a valuable clinical tool for neurosurgeons in patient selection. Based on our experience from all operations, we developed this grading system by giving points to each parameter according to its difficulty level.

This grading system can be easily applied by assigning points to the 5 parameters and generating scores from 0 to 10 (Table 6). As the grade increases, the difficulty for clipping contralateral aneurysm also increases. To our experience, the contralateral aneurysm can clip with low difficulty in grades 0 to 1 of this proposed system and with intermediate difficulty in grades 2 to 3. If total grade score is between 4 and 6, difficulty is high grade and one must be very careful when deciding on a unilateral approach. If total grade score is between 7 and 10, difficulty is very high grade and a unilateral approach should not be attempted. As can be understood from our grading system, suitable candidates for the unilateral approach are quite limited.

In order to test the predictive value of this grading system, in the second part of this series (2011–2023), patients were selected considering these 5 parameters, and 20 more patients with bMCA aneurysm were operated on using unilateral approach by senior author. Contralateral MCA aneurysm was clipped in 19 patients, except one (95%). The difference between the first and second parts of this study in the clipping rate of contralateral aneurysms (10 of 22 and 19 of 20, respectively) was highly statistically significant (p = 0.001, Pearson Chi Square test).

In the microsurgical anatomical study of Oshiro et al. published in 1997, [24] exposure of contralateral MCA bifurcation using unilateral approach was possible in 62% (10 of 16) of cadaver specimens. This rate was 65.2% in case series of de Sousa et al. [10]. In the second part of our study (2012–2023), we could be clipped the contralateral aneurysm with a unilateral approach in 95% of the patients. Of course, our high success rate must be due to the fact that we selected our patients according to these parameters in the second part of the study.

Unfortunately, since there is no other grading system on this subject in the literature, we cannot compare advantages and disadvantages of our own grading system with others.

Of course, one of the most important factors for success in these challenging cases is the experience of the neurosurgeon. However, experience is not a measurable factor. Therefore, a fully objective evaluation cannot be made on this subject. However, we can make a simple assessment: based on our own experience, a neurosurgeon should not attempt unilateral approach without performing at least 250–300 standard aneurysm surgeries, as we stated in our previous report [17].

#### Endovascular treatment

Significant advances have been made in endovascular therapy in the last two decades. Successful results have also been achieved in the treatment of MCA aneurysms [12, 18]. Stent + coiling and/or flow diversion methods has some advantages over the surgical clipping: 1) It is less invasive technique than clipping, 2) It offers the advantage of avoiding aneurysm manipulation that increases the rupture risk, 3) Recovery time of the patient is shorter. But, endovascular methods are still not an ideal choice for MCA aneurysms due to their often complex aneurysm and/or vessel configurations and broad necks. The majority of the publications in the last few years indicate that surgical treatment is still superior in MCA aneurysms in terms of higher complete occlusion rate, longer durability, and lower periprocedural thromboembolism rate [8, 11, 14, 22, 26, 33].

Endovascular treatment also has some disadvantages: 1) if intraprocedural rupture occurs, its control is much more difficult compared with microsurgical treatment; 2) the use of stents or flow diverters will necessarily require long-term antiplatelet therapy (at least 3–6 months), 3) radiation exposure is higher than single aneurysm cases, 4) need for repeated imaging examinations, 5) other general disadvantages are also high recurrence rates, unknown durability over the long term 6) After the procedure, complete resolution of the aneurysm can take a long time. During this period of time, the most important risk is delayed hemorrhages. On the other hand, in open surgery of bilateral MCA aneurysms, the source of bleeding can be precisely determined visually. This is not possible in endovascular treatment. In this situation, if the ruptured aneurysm is not correctly identified, the ruptured aneurysm will remain untreated and the patient will potentially be at risk of rebleeding.

However, endovascular treatment should be preferred in the following situations: 1) inappropriate surgical parameters (severe brain edema, long A1 + M1 segment, irregular shape), 2) severe medical comorbidities, 3) poor neurological status, 4) the patient’s preference for endovascular treatment, and 5) insufficient experience of the neurosurgeon. As a matter of fact, in the second part of this study (2011–2023), endovascular treatment was recommended for 4 patients with bMCA aneurysms.

## Limitation of this study

This study has some limitations: 1) This is a retrospective review of a single vascular neurosurgeon’s experience more than 20-years period. Therefore, these results may not be generalizable to other less specialized neurosurgeons, 2) The parameters were chosen based on surgical difficulties we personally encountered throughout this series. Other neurosurgeons may find some parameters unnecessary or add new parameters, 3) This grading system was developed by a single neurosurgeon and its validity was also evaluated by him. Therefore, the experience of other neurosurgeons is needed to evaluate its validity more objectively, 4) Although this is the second largest surgical series in the literature with 31 cases, the number of cases is not very large. However, considering that the largest published series consists of 38 cases [2], it is clear that it is not easy to reach larger series, 5) Longer follow-up periods are necessary for definitive conclusions about the long-term efficacy of this approach.

## Strengths of this study

We think this study has two strengths: 1) This is the second largest series in the literature on this subject, 2) For the first time in the literature, a grading system that preoperatively evaluates the difficulties of the unilateral approach to bilateral MCA aneurysms is presented.

## Conclusions

Patients with bMCA aneurysms can be operated via unilateral approach in experienced hands in properly selected cases. The proposed grading system may help the neurosurgeon in this selection by preoperatively predicting intraoperative difficulties.

## Data Availability

No datasets were generated or analysed during the current study.

## Abbreviations

bMCA: Bilateral Middle Cerebral Artery
DSA: Digital Subtraction Angiography
ICA: Internal Carotid Artery
MRI: Magnetic Resonance Imaging
mRS: Modified Rankin Scale
3D-CTA: Three Dimensional Computed Tomography Angiography
